# Mice with a Mutation in the *Mdm2* Gene That Interferes with MDM2/Ribosomal Protein Binding Develop a Defect in Erythropoiesis

**DOI:** 10.1371/journal.pone.0152263

**Published:** 2016-04-04

**Authors:** Takuya Kamio, Bai-wei Gu, Timothy S. Olson, Yanping Zhang, Philip J. Mason, Monica Bessler

**Affiliations:** 1 Department of Hematology, The Children's Hospital of Philadelphia, Philadelphia, PA, United States of America; 2 Comprehensive Bone Marrow Failure Center, The Children's Hospital of Philadelphia, Philadelphia, PA, United States of America; 3 Department of Radiation Oncology and Lineberger Comprehensive Cancer Center, The University of North Carolina at Chapel Hill School of Medicine, Chapel Hill, NC, United States of America; Rutgers-Robert wood Johnson Medical School, UNITED STATES

## Abstract

MDM2, an E3 ubiquitin ligase, is an important negative regulator of tumor suppressor p53. In turn the *Mdm2* gene is a transcriptional target of p53, forming a negative feedback loop that is important in cell cycle control. It has recently become apparent that the ubiquitination of p53 by MDM2 can be inhibited when certain ribosomal proteins, including RPL5 and RPL11, bind to MDM2. This inhibition, and the resulting increase in p53 levels has been proposed to be responsible for the red cell aplasia seen in Diamond-Blackfan anemia (DBA) and in 5q- myelodysplastic syndrome (MDS). DBA and 5q- MDS are associated with inherited (DBA) or acquired (5q- MDS) haploinsufficiency of ribosomal proteins. A mutation in *Mdm2* causing a C305F amino acid substitution blocks the binding of ribosomal proteins. Mice harboring this mutation (*Mdm2*^*C305F*^), retain a normal p53 response to DNA damage, but lack the p53 response to perturbations in ribosome biogenesis. While studying the interaction between RP haploinsufficiency and the *Mdm2*^*C305F*^ mutation we noticed that *Mdm2*^*C305F*^ homozygous mice had altered hematopoiesis. These mice developed a mild macrocytic anemia with reticulocytosis. In the bone marrow (BM), these mice showed a significant decrease in Ter119^hi^ cells compared to wild type (WT) littermates, while no decrease in the number of mature erythroid cells (Ter119^hi^CD71^low^) was found in the spleen, which showed compensated bone marrow hematopoiesis. In methylcellulose cultures, BFU-E colonies from the mutant mice were slightly reduced in number and there was a significant reduction in CFU-E colony numbers in mutant mice compared with WT controls (*p* < 0.01). This erythropoietic defect was abrogated by concomitant p53 deficiency (*Trp53*^*ko/ko*^). Further investigation revealed that in *Mdm2*^*C305F*^ animals, there was a decrease in Lin^-^Sca-1^+^c-Kit^+^ (LSK) cells, accompanied by significant decreases in multipotent progenitor (MPP) cells (*p* < 0.01). Competitive BM repopulation experiments showed that donor BM harboring the *Mdm2*^*C305F*^ mutation possessed decreased repopulation capacity compared to WT BM, suggesting a functional stem cell deficit. These results suggest that there is a fine tuned balance in the interaction of ribosomal proteins with the MDM2/p53 axis which is important in normal hematopoiesis.

## Introduction

Diamond-Blackfan Anemia (DBA) is a bone marrow failure syndrome characterized by macrocytic anemia and red cell aplasia, sometimes associated with developmental anomalies, chiefly cleft palate, thumb abnormalities and cardiovascular problems [1, 2]. Patients are usually diagnosed in their first year and can be treated with steroids or blood transfusion. In at least 70% of cases the disease is due to an inherited mutation in one of several ribosomal proteins, though the mechanism whereby haploinsufficiency for ribosomal proteins, which are required in all dividing cells, leads to specific problems with erythropoeisis, has not been elucidated [3]. In acquired 5q- MDS, patients also have a defect in erythroid cell differentiation with macrocytic anemia and red cell aplasia [4]. The gene for a ribosomal protein RPS14 is present in the deleted region and appears to be responsible for the development of the red cell defect [5]. In animal and cellular models, defects in red cell production caused by ribosomal protein deficiencies are rescued by the absence of p53, which responds to cellular signals and controls progression through cell cycle checkpoints [6–9]. p53 is regulated by the E3 ubiquitin ligase MDM2, which ubiquitinates p53 stimulating its turnover within the nucleus [10–15]. The *Mdm2* gene is a transcriptional target of p53 thus forming a feedback loop that controls p53 and MDM2 levels [16]. Certain ribosomal proteins, notably RPL5 and RPL11, bind to MDM2 and block its ability to inhibit p53. This mechanism was demonstrated by the inability of ribosome biogenesis to affect p53 in cells homozygous for a mutant MDM2 with a C305F mutation, which fails to bind these ribosomal proteins (RPs) [17]. Cells homozygous for *Mdm2*^*C305F*^ have a normal p53 response to DNA damage but not to ribosomal stress.

A crucial stage in mammalian erythropoiesis takes place in erythroblasts, rapidly proliferating cells with a maximum rate of ribosome production, needed for globin synthesis. The co-ordination between ribosome synthesis and cell division must be closely regulated in these cells, given the abortive effects on erythropoiesis when ribosome synthesis is perturbed. We therefore hypothesized that the RP/MDM2/p53 axis may play a crucial role in normal erythropoiesis. To test our hypothesis we studied erythropoiesis in homozygous *Mdm2*^*C305F*^ mice compared to wild type (WT) mice. We found that *Mdm2*^*C305F*^ mice had distinct abnormalities in erythropoiesis that point to the importance of RP/MDM2 binding in regulating red cell production.

## Materials and Methods

### *Mdm2* mutant mice and animal experiments

We obtained mice carrying *Mdm2*^*C305F*^ mutation from Dr. Zhang [18], and *Trp53*^*ko*^ (129S2/SvPas) from the Jackson Laboratory. Animals were interbred to maintain *Mdm2*^*C305F*^, which was bred to C57BL/6 mice for studies. All mice were handled in strict accordance with our protocol (IACUC 911) as approved by the institutional animal care and use committee at the Children’s Hospital of Philadelphia Animal Care Facility.

Peripheral Blood cell counts were performed using a Hemavet HV950FS analyzer (Drew Scientific). Absolute reticulocyte counts and erythrocyte adenosine deaminase (eADA) were performed as previously described [19, 20]. For erythropoietin, lactate dehydrogenase (LDH), and bilirubin serum levels, serum from the mice was collected by centrifugation of whole peripheral blood at 1,000 *g* for 20 minutes. Erythropoietin levels in serum were determined by ELISA assay kit (R&D Systems) [21]. LDH and bilirubin in serum were analyzed used a Vistros 350 chemistry system (Ortho Clinical).

### Histology and immunohistochemistry

Mouse peripheral blood smears were stained with May-Grunwald (Sigma-Aldrich) and Gimsa (Sigma-Aldrich) reagents. Mouse bone marrow and spleen tissue were fixed in 10% formalin. Bones were decalcified before dehydration, paraffin-embedded, and cut into 5 μm sections. For immunohistochemistry, Ter119 antibody (BD Biosciences) was used to stain formalin fixed paraffin embedded TMA slides. The sections were then incubated with TER-119 antibody at a 1:50 dilution for 1 hour at room temperature and then were incubated with biotinylated anti-rat IgG (Vector Laboratories) for 30 min at room temperature. After rinsing, sections were then incubated with the avidin biotin complex (Vector Laboratories) for 30 min at room temp. The sections were then rinsed and incubated with DAB (DAKO Cytomation) for 10 min at room temperature. The sections were also counterstained with hematoxylin and eosin (Sigma-Aldrich), rinsed, dehydrated in ethanol followed by xylene, then coverslipped. The preparations were examined by light microscopy (BX43; Olympus).

### Flow cytometric measurements

For assessment of bone marrow and spleen progenitor cells by flow cytometry, single-cell suspensions of freshly prepared bone marrow and spleen were incubated with the following anti-mouse monoclonal antibodies: Ter119 (Ly-76, BD Biosciences), CD71 (C2, BD Biosciences), CD34 (RAM34, BD Biosciences), CD135 (A2F10, eBioscience), CD127 (A7R34, eBioscience), c-Kit (ACK2, eBioscience), SCA-1 (LY-6A/E, BD Biosciences), eFluor520 (eBioscience), and 7-AAD (eBioscience). Lineage (lin) staining included anti-mouse Ter119 (BD Biosciences), GR-1 (RB6-8C5, BD Biosciences), B220 (RA3-6B2, BD Biosciences), CD4 (RM4-5, BD Biosciences), CD8 (53–6.7, BD Biosciences), and CD11b (M1/70, BD Biosciences) antibodies [22–24]. Multicolor data acquisition for bone marrow progenitor subsets was performed on a FACSCanto II flow cytometer, and data were analyzed with FlowJo 7.6.1.

### Colony-forming cell assays

Bone marrow (BM) cells were plated in triplicate in Methocult M3334 (StemCell Technologies) into 35 mm dishes at the density of 2 x 10^5^ cells per dish and cultured for 2 days (CFU-E) or 4 days (mature BFU-E). BM cells were plated in triplicate in Methocult M3434 (StemCell Technologies) into 35 mm dishes at the density of 2 x 10^5^ or 2 x 10^4^ cells per dish and cultured for 7 days (BFU-E and CFU-GEMM) or 12 days (CFU-GM) [25]. Cells were stained with Benzidine dihydrochloride (Sigma-Aldrich) solution and visible colonies were counted.

### Bone marrow transplantation and long-term repopulating assay

Competitive repopulation assays were performed by injecting 2.5×10^6^ bone marrow cells from either experimental (*Mdm2*^*C305F/C305F*^) or control (*Mdm2*^*C305F/+*^, C57BL/6) donor animals on a CD45.2 background, along with 2.5×10^6^ competitor cells from CD45.1 C57BL/6 mice into irradiated (900 cGy) C57BL/6 recipient mice (CD45.1) [26, 27]. Peripheral blood samples were collected at 4, 8, and 12 weeks following transplant to assess for the percentage of reconstitution of peripheral blood cell lineages with CD45.2 cells, thus defining relative repopulation capacity of bone marrow harvested from our experimental versus control mouse strains in each lineage analyzed. Leukocyte subsets were assessed after RBC lysis with ammonium chloride, using the following antibodies: FITC antibodies against, B220 (RA3-6B2, BD Biosciences), TCR β (H57-597, BD Biosciences), CD45.2 (104, BD Biosciences), and CD4 (GK1.5, BD Biosciences); PE antibodies against, CD11b (M1⁄70, BD Biosciences), Gr-1 (RB6-8C5, BD Biosciences), CD8 (53–6.7, eBioscience), and CD45.1 (A20, BD Biosciences).

### Statistical analysis

Data were analyzed for statistical significance with the 2-tailed paired Student’s *t*-test (*p < 0.05;**P < 0.01, Excel 2013, Microsoft Corporation) for 2-group comparisons. Error bars were generated using the standard error of the mean.

## Results

### Hematologic characterization in *Mdm2*^*C305F/C305F*^ mutant mice

To examine our hypothesis that co-ordination of ribosome biogenesis and the cell cycle is important in hematopoiesis we first studied peripheral blood counts in mice homozygous for *Mdm2*^*C305F/C305F*^. These mice displayed a mild macrocytic anemia with reticulocytosis (Table 1). Red cell morphology, other than a mild macrocytosis, was normal and there were no signs of hemolysis. Erythropoietin levels were higher in mutant mice compared to wild type, but this did not reach statistical significance (*p* = 0.118). eADA levels, known to be increased in red blood cells from patients with DBA due to ribosomal protein gene mutations, were normal in red blood cells from *Mdm2*^*C305F/C305F*^ mutant mice. White blood cells and platelets were not significantly different between mutant and control mice. The anemia correlated with *Mdm2*^*C305F*^ gene dosage with the homozygous mice exhibiting lower levels of hemoglobin and number of red blood cells than the heterozygous mice.

**Table 1:** Effects of *Mdm2*^*C305F*^ mutant on blood counts, lactate dehydrogenase, bilirubin, erythropoietein level, and eADA activity.

	*C57BL/6*	*Mdm2*^*C305F/+*^	*Mdm2*^*C305F/C305F*^	*Mdm2*^*C305F/C305F*^*;Trp53*^*ko/ko*^
No. of animals	13	12	12	14
WBC(x10^3^/μL)	7.19 ± 2.30	6.73 ± 1.54	6.27 ± 1.91	7.46 ± 1.65
Neutrophil(x10^3^/μL)	0.69 ±0.28	0.89 ± 0.42	0.78 ± 0.24	1.51 ± 0.34 ^a^§
Lymphocyte(x10^3^/μL)	6.94 ± 1.96	5.60 ± 1.33	5.26 ± 1.65	5.70 ± 1.43
Monocyte(x10^3^/μL)	0.27 ± 0.12	0.23 ± 0.07	0.20 ± 0.06	0.23 ± 0.09
Eosinophil(x10^3^/μL)	0.02 ± 0.02	0.02 ± 0.01	0.03 ± 0.02	0.01 ± 0.01 ^c^§
Basophil(x10^3^/μL)	0.00 ± 0.01	0.01 ± 0.01	0.00 ± 0.01	0.00 ± 0.01
RBC(x10^6^/μL)	11.45 ± 0.59	10.68 ± 0.93	9.87 ± 1.03 ^a^†	10.05 ± 0.50
Hb(g/dL)	14.74 ± 0.75	13.58 ± 1.00	13.53 ± 1.19 ^b^†	13.10 ± 0.79
MCV(fL)	51.89 ± 4.05	54.83 ± 4.14	58.88 ± 3.79 ^a^†	52.66 ± 0.94 ^a^§
Reticulocyte(‰)	3.09 ± 0.81	4.05 ± 0.93	4.64 ± 1.29 ^b^†	4.89 ± 0.94
Platelets(x10^3^/μL)	729.54 ± 113.81	722.08 ± 134.73	695.50 ± 86.27	812.00 ± 80.29 ^c^§
No. of animals	7	7	6	4
LDH(IU/L)	522.71 ± 60.2	505.00 ± 53.42	565.67 ± 95.28	617.8 ± 159.01
T-Bil(mg/dL)	0.39 ± 0.12	0.39 ± 0.14	0.43 ± 0.05	0.40 ± 0.12
No. of animals	17	12	13	15
Epo(pg/ml)	168.73 ± 46.60	169.07 ± 44.40	204.93 ± 71.78	168.71 ± 66.43
No. of animals	9	8	12	5
eADA(IU/g Hb)	1.15 ± 0.18	1.16 ± 0.16	1.16 ± 0.22	1.11 ± 0.16
No. of animals	15	8	8	9
Body weight(g)	27.60 ± 4.48	29.27 ± 3.10	26.27 ± 3.48	29.17 ± 4.21
No. of animals	15	8	8	13
Bone marrow cells(x10^7^ cells)	4.534 ± 0.561	4.337 ± 0.870	3.609 ± 0.770 ^b^†	3.925 ± 0.591
No. of animals	21	9	9	12
Spleen(mg)	79.5 ± 13.2	84.1 ± 14.2	100.3 ± 15.2 ^b^†	107.0 ± 29.3

Blood counts analyses of mice at 6 weeks of age. Bone marrow cellularity determined by flushing 2 femurs and 2 tibias. WBC indicates white blood cell count; RBC, red blood cell count; Hb, hemoglobin; MCV, mean corpuscular volume; LDH, lactate dehydrogenase; T-bil, total bilirubin; Epo, erythropoietin; and eADA, erythrocyte adenosine deaminase activity. All values are given as mean ± standard error of mean, two-tailed Student’s *t-test*,

^a^*P* < 0.001,

^b^*P* < 0.01,

^c^*P* < 0.05.

^†^ C57BL/6 vs *Mdm2*^*C305F/C305F*^

^§^ *Mdm2*^*C305F/C305F*^ vs *Mdm2*^*C305F/C305F*^*;Trp53*

We also found that homozygous mutant *Mdm2*^*C305F*^ animals had a decreased number of nucleated cells in bone marrow and splenomegaly compared to control animals (Table 1). *Mdm2*^*C305F*^ homozygous and heterozygous mice were indistinguishable from wild type mice with no noticeable differences in appearance and body weight. Animals were healthy and during the observation period of 12 months no tumors were observed in mutant or wild type mice.

**Fig 1:**
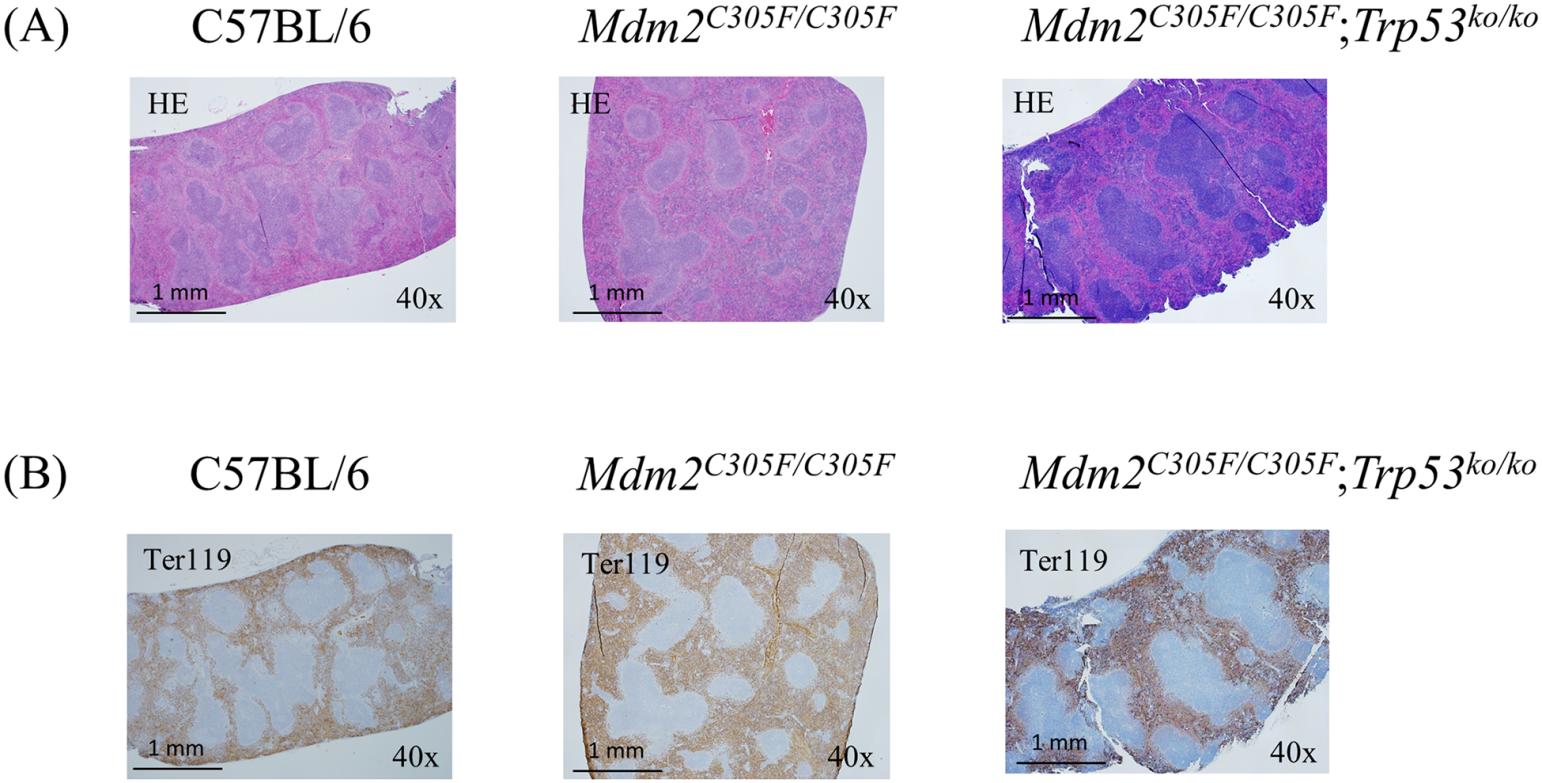
Effects of *Mdm2*^*C305F*^ mutant on peripheral blood, bone marrow, and spleen histology. (A) Hematoxylin and eosin stained splenic sections (Magnification: 40x). (B) Ter119-stained (brown) splenic sections. Blue is counterstained lymphoid tissue.

The cellular makeup of *Mdm2*^*C305F*^ homozygous spleens was determined by comparative histology and immunophenotyping using flow cytometry. Germinal centers were histologically normal, but the red pulp was increased (Fig 1), with increased staining for glycophorin-associated Ter119, a marker of erythroid cells.

**Fig 2:**
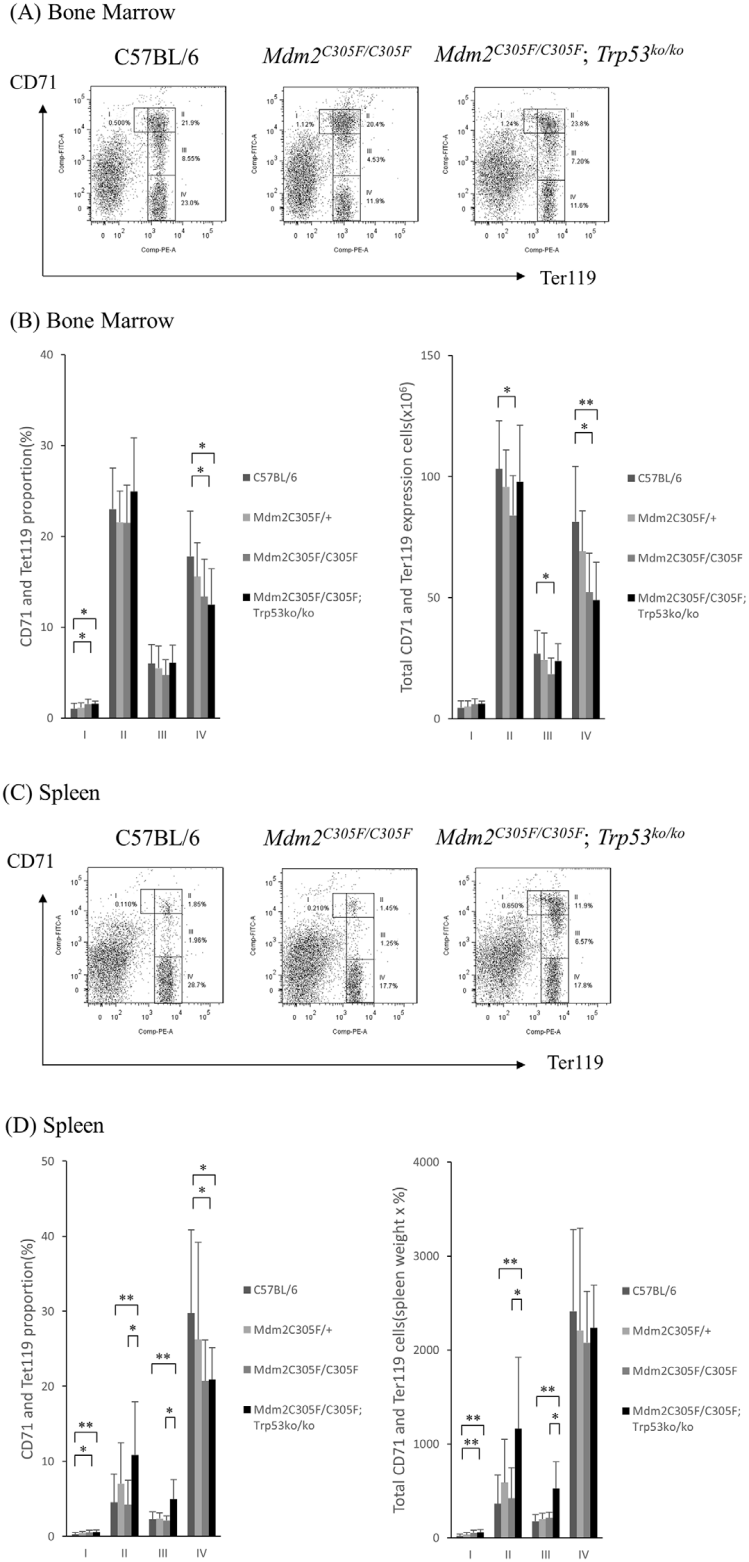
Effects of *Mdm2*^*C305F*^ mutant on erythroid maturation in bone marrow and spleen. Flow cytometric assessment of cell-surface Ter119 and CD71 expression in bone marrow and spleen, used to define population of proerythroblasts (I; Ter119^med^CD71^hi^), basophilic erythroblasts (II; Ter119^hi^CD71^hi^), chromatophilic erythroblasts (III; Ter119^hi^CD71^med^), and orthochromatic erythroblasts (IV; Ter119^hi^CD71^low^). Specific populations in control (n = 25), *Mdm2*^*C305F*^ heterozygous (n = 9), *Mdm2*^*C305F*^ homozygous (n = 8), and *Mdm2*^*C305F/C305F*^*;Trp53*^*ko/ko*^ mice (n = 8). (A) Representative flow cytometry analysis of bone marrow. (B) *Mdm2*^*C305F*^ homozygous mice showed a significant decrease in Ter119^high^ cells compared to wild type littermates in the bone marrow. (C) Representative flow cytometry analysis of spleen. (D) *Mdm2*^*C305F*^ homozygous mice showed no decrease in the number of mature erythroid cells (Ter119^hi^CD71^low^) compared to wild type littermates in the spleen.

In the bone marrow *Mdm2*^*C305F*^ homozygous mice showed a significant decrease in Ter119^high^ cells compared to wild type littermates as measured by flow cytometry. In contrast, while the spleen contained a decreased percentage of Ter119^high^ cells, the total number of mature erythroid cells (Ter119^hi^CD71^low^) in the spleen was not decreased. This discrepancy between erythroid cell percentage and absolute number in the spleen was due to the splenomegaly and expanded red pulp seen in *Mdm2*^*C305F*^ homozygous mice, which is consistent with compensatory increased extramedullar (splenic) erythropoiesis in these mice (Fig 2).

**Fig 3:**
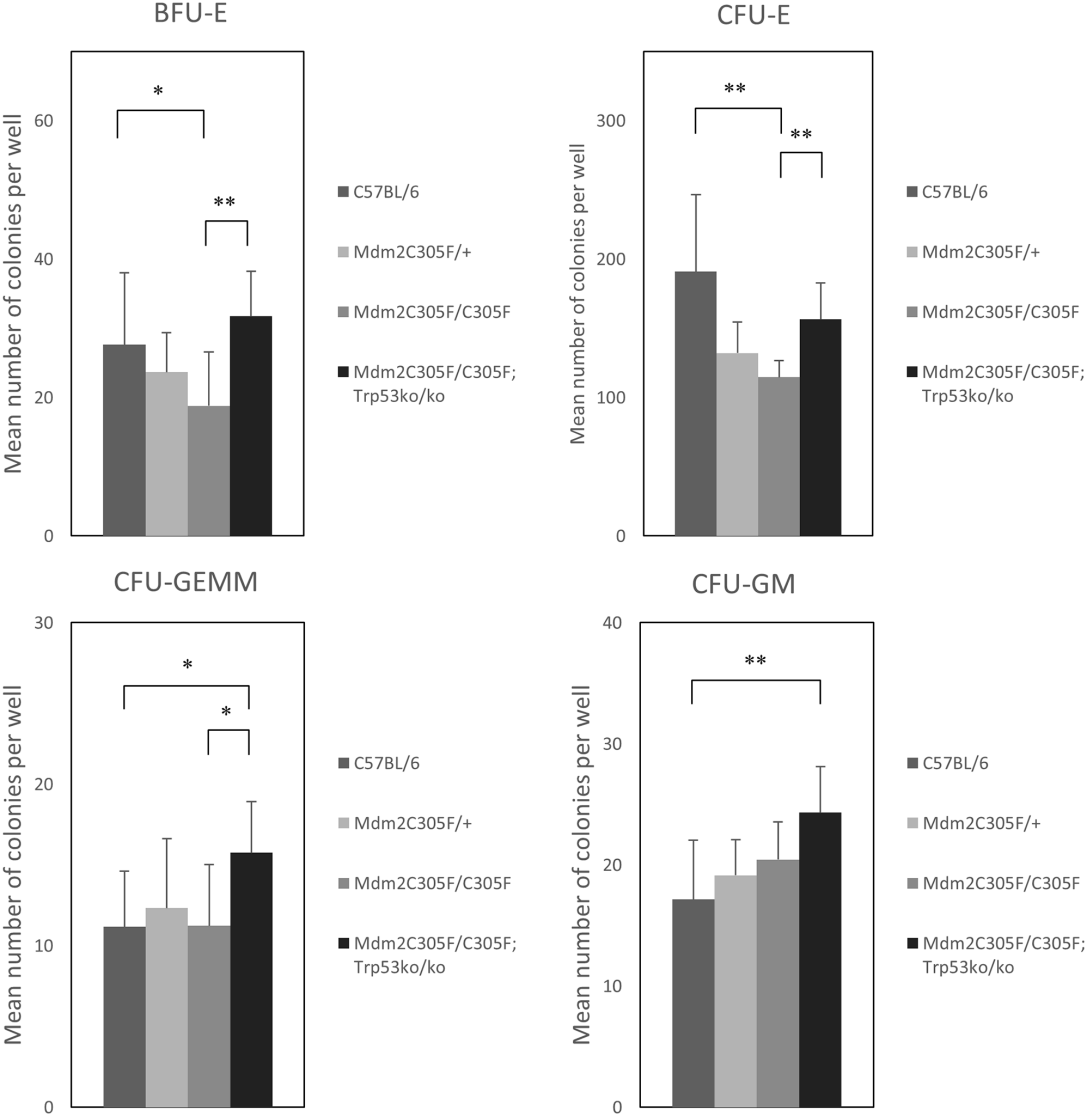
Hematopoietic colony assays in *Mdm2*^*C305F*^ mutant mice. Hematopoietic colony assays in C57BL/6 (n = 14), *Mdm2*^*C305F*^ heterozygous (n = 7), *Mdm2*^*C305F*^ homozygous (n = 7), and *Mdm2*^*C305F/C305F*^;*Trp53*^*ko/ko*^ (n = 7) mice. Mean ± standard error of mean, two-tailed Student’s *t*-test (*P < 0.05, **P < 0.01). BFU-E colonies from the mutant mice were slightly reduced in number and there was a significant reduction in CFU-E colony numbers in mutant mice compared with wild type (WT) controls.

In methyl cellulose cultures BFU-E colonies from the mutant mice were slightly reduced in number and there was an even greater reduction in CFU-E colony numbers in mutant mice compared with WT controls (*p* < 0.001) (Fig 3). CFU-GEMM and CFU-GM were not significantly different between mutant and control mice (Fig 3).

### *Mdm2*^*C305F/C305F*^ mutant mice are defective in stem cell renewal

**Fig 4:**
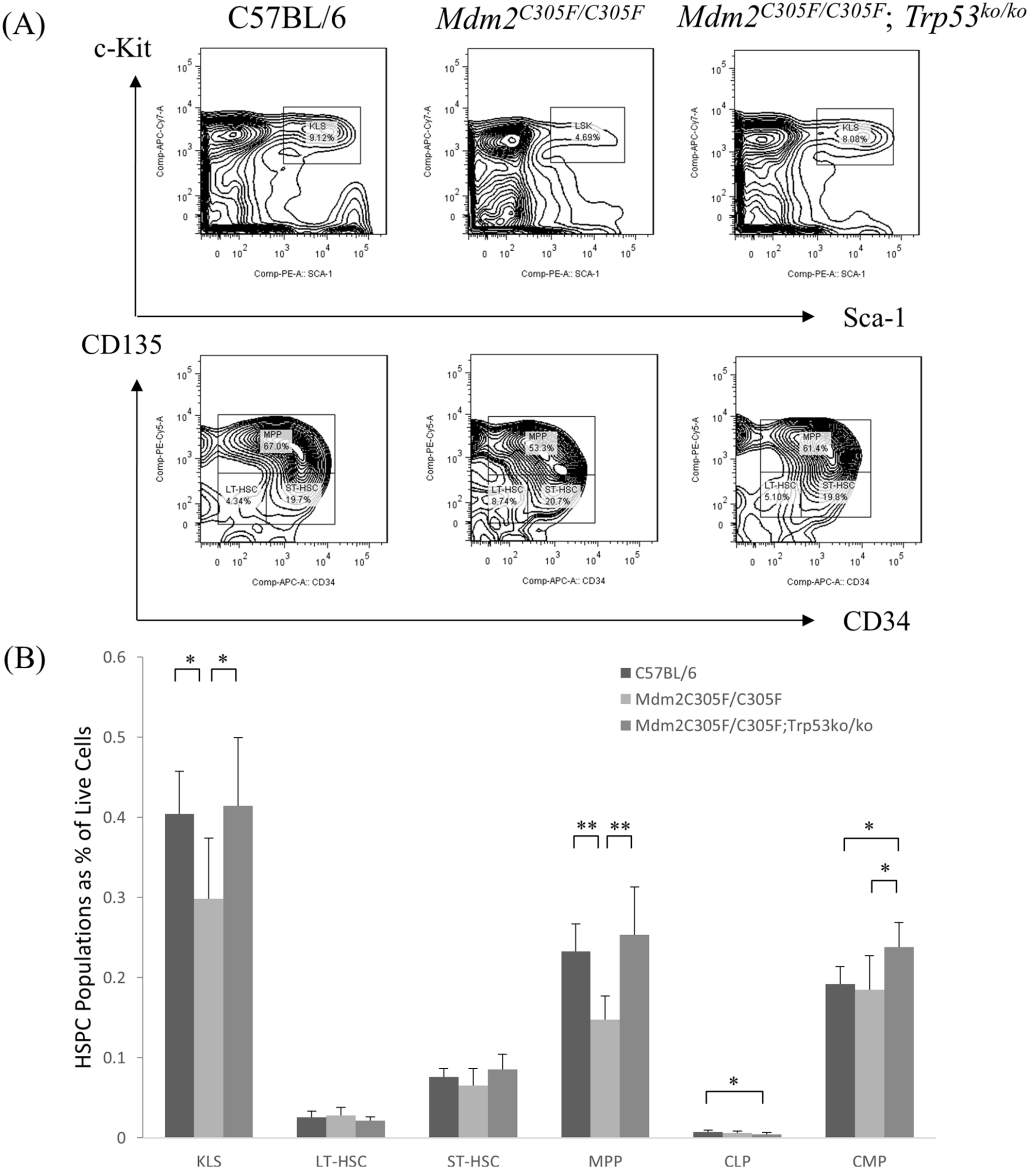
Effects of *Mdm2*^*C305F*^ mutant on selected hematopoietic lineage subpopulation in bone marrow. (A) Representative flow cytometry analysis of the specific precursor populations in control (n = 7), *Mdm2*^*C305F*^ homozygous (n = 6), and *Mdm2*^*C305F/C305F*^;*Trp53*^*ko/ko*^ mice (n = 6). (B) *Mdm2*^*C305F*^ mutant animals exhibited a significant paucity in the HSPC population of LSKs (*P* < 0.01), accompanied by significant decreases of MPP populations (*P* < 0.01). HSPC indicates hematopoietic stem and progenitor cell; LSK, Lin^-^Sca-1^+^c-Kit^+^; LT-HSC, long-term hematopoietic stem cells; ST-HSC, short-term hematopoietic stem cells; MPP, multipotent progenitor; CLP, common lymphoid progenitor; and CMP, common myeloid progenitor.

To explore the ontogeny of the erythroid defects caused by the *Mdm2*^*C305F*^ mutation in the mouse, we analyzed specific precursor populations in the bone marrow cells. *Mdm2*^*C305F*^ mutant animals exhibited a significant decrease in Lin^-^Sca-1^+^c-Kit^+^ (LSK) cells, accompanied by significant decreases in multipotent progenitor (MPP) cells (*p* < 0.01) (Fig 4).

Next, we were interested in whether the abnormality in *Mdm2*^*C305F*^ mutant occurs during hematopoietic differentiation or also at the stem cell level. For this, competitive BM transplant experiments were performed using a 50:50 mixture of BM cells from either *Mdm2*^*C305F*^ homozygous or heterozygous mutant mice and WT mice all at 8 weeks of age. After primary transplantation BM cells from *Mdm2*^*C305F*^ homozygous mice contributed only little to circulating B and T lymphocytes compared to WT derived BM cells, whereas the contribution to the myeloid lineage in peripheral blood at day 28 after transplant was approximately equal to WT competitor cells but decreased with time after transplant (S1 Fig). In contrast, for *Mdm2*^*C305F*^ heterozygous and WT BM cells the contribution was higher and remained stable during the observation period suggesting, a defect at an earlier precursor, possibly at a stem cell level.

The results from competitive BM transplant experiments indicate that *Mdm2*^*C305F*^ homozygous BM cells have an intrinsic proliferative defect at an early progenitor level as well and possibly more pronounced during erythroid differentiation compared to *Mdm2*^*C305F*^ heterozygous or WT mice.

### Effects of *Mdm2*^*C305F/C305F*^ are partially corrected by *Trp53*^*ko/ko*^

We next asked whether *p53* was required for the observed hematologic changes in the *Mdm2*^*C305F*^ mutant mouse. For this *Mdm2*^*C305F*^ animals were crossbred with mice carrying a *p53* knockout allele, to generate mice homozygous for *Mdm2*^*C305F*^ *and Trp53*^*ko*^. In *Mdm2*^*C305F*^ animals, deficiency for p53 (*Trp53*^*ko/ko*^) mitigated the increase in mean corpuscular volume (MCV) at 6 weeks of age, although the RBC count and Hemoglobin did not significantly increase and reticulocytosis persisted (Table 1). Double homozygous *Mdm2*^*C305F/C305F*^;*Trp53*^*ko/ko*^ mice had a persistent splenomegaly with a significant increase in basophilic and chromatophilic erythroblasts (Fig 2). Furthermore, BFU-E's, CFU-E's, CFU-GEMM's, and MPP's from the *Mdm2*^*C305F/C305F*^;*Trp53*^*ko/ko*^ mice were significantly increased in number compared to *Mdm2*^*C305F*^ mutant mice (Figs 3 and 4). This suggests that the loss of p53 rescues impaired mutant *Mdm2*^*C305F*^ erythropoiesis at least in part. *Mdm2*^*C305F/C305F*^;*Trp53*^*ko/ko*^ mice showed also a significant increase in the number of neutrophil and platelets, however this was not different to the neutrophil and plated counts in p53 mutant mice thus likely caused by the *p53* knockout background [28].

## Discussion

DBA and 5q- MDS are associated with inherited and acquired haploinsufficiency of ribosomal proteins. Recent studies have implicated the role of p53 in mediating a cellular response to abnormal ribosome biogenesis and a ribosome deficient animal model showed p53-dependent abnormalities in development and hematopoiesis [29].

Oncogene-induced p53 activation is a major tumor-suppressor mechanism [10–12]. MDM2 is an important negative regulator of p53 that is important in cell cycle control. Recent study has demonstrated that *Mdm2*^*C305F*^ mutation disrupted ribosomal protein binding and exhibited a normal p53 response to DNA damage but failed to activate p53 after perturbations in ribosome biogenesis [17].

**Fig 5:**
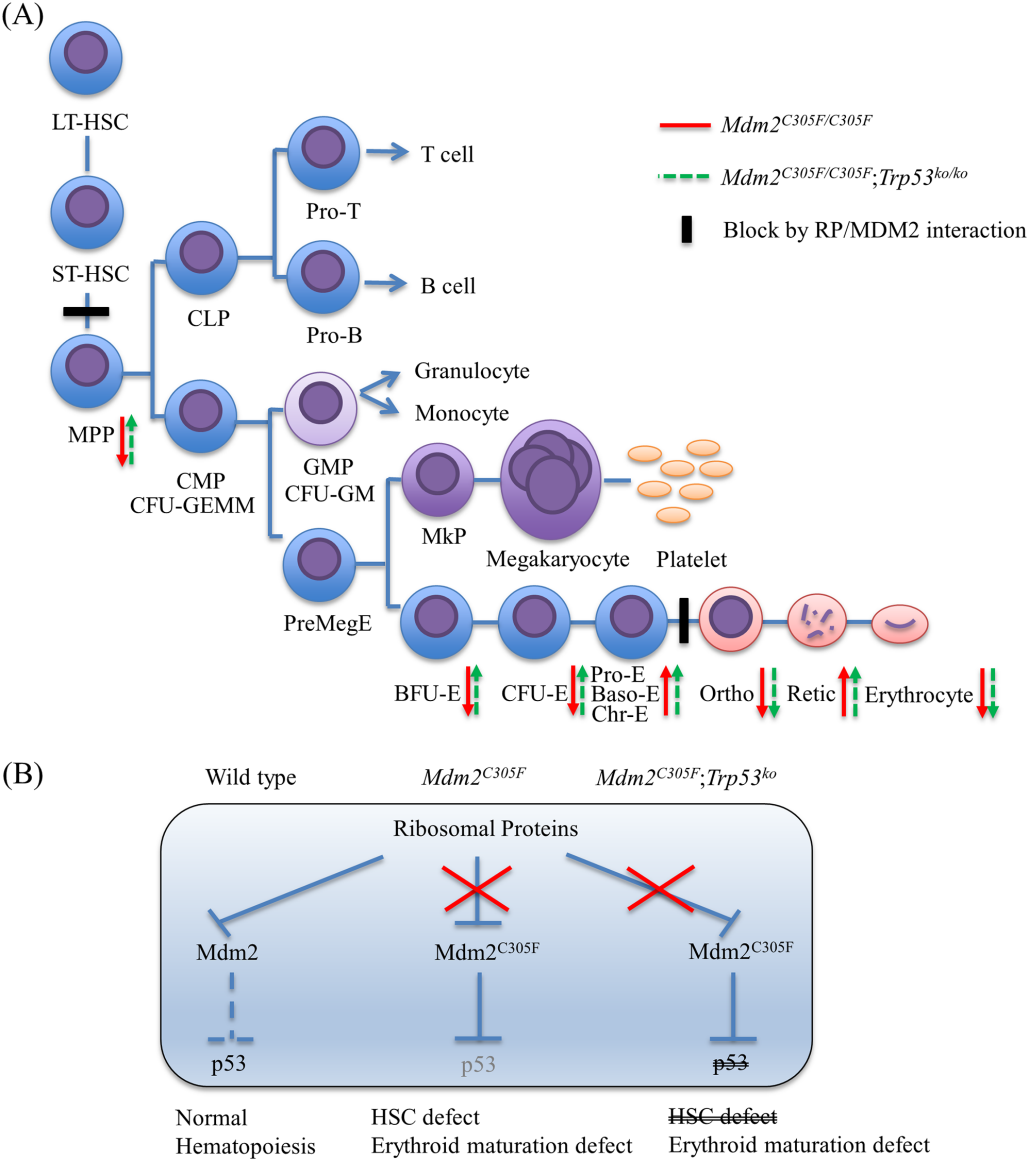
Diagram of the hematopoietic lineage and differences observed in *Mdm2*^*C305F*^ mutant mice. (A) *Mdm2*^*C305F*^ phenotype corresponded to blocks at MPP and orthochromatic erythroblast. LSK indicates Lin^-^Sca-1^+^c-Kit^+^; LT-HSC, long-term hematopoietic stem cells; ST-HSC, short-term hematopoietic stem cells; MPP, multipotent progenitor; CLP, common lymphoid progenitor; CMP, common myeloid progenitor; GMP, granulocyte/macrophage progenitor; PreMegE, megakaryocyte–erythroid progenitor; and MKP, megakaryocyte progenitor. (B) RP/MDM2 interaction may be important for the mature hematopoiesis rather than for the HSC proliferation.

While studying the interaction between RP haploinsufficiency, we noticed that *Mdm2*^*C305F*^ mutant mice themselves had perturbed hematopoiesis. These mice showed a dramatic decrease in progenitor cells and a delayed erythroid differentiation without obvious myeloid and lymphoid lineages defects (Fig 5), a phenotype reminiscent to the defect described in CD34^+^ cells from DBA patients with inherited *RPL11* mutations, underlining the importance of the co-ordination of ribosome biogenesis with cell cycle control in erythropoiesis[30].

Similar to the experiments using RPL11 deficient human CD34^+^ cells from patients with DBA, *p53* knockout in our *Mdm2* mutant animals partially mitigated the decrease in progenitor cells and the delayed hematopoiesis in our mice. However, although the progenitor numbers from *Mdm2*^*C305F*^ mutant mice were mitigated by *p53* knockout the animals were still anemic although they lost the *Mdm2*^*C305F*^ associated macrocytosis, suggesting that the half-life of the mature *Mdm2*^*C305F*^ *p53* mutant red cells is decreased. Indeed double mutant *Mdm2*^*C305F*^ *p53* knockout mice had slightly elevated LDH levels and the accumulation of iron in their spleen (Table 1, S2 Fig).

Previous studies showed that MDM2 deficiency in mice results in embryonic lethality due to massive apoptosis and this phenotype was rescued by the *p53*^*515C*^ allele, which encodes an apoptosis-deficient protein [16]. These mice died early, due to a severe impairment of progenitor cell expansion during postnatal hematopoiesis, leading to p53-dependent cell cycle arrest. These results show the regulation of p53 by MDM2 is important in hematopoiesis. In contrast to these studies, our *Mdm2*^*C305F*^ mutant mice show similar survival to wild type [17].

Very recently the *RPS19*-deficient DBA with *Mdm2*^*C305F*^ knock-in mice were published[31]. Mdm2^C305F^ improved expansion of hematopoietic progenitors, but the extent of the erythroid rescue by Mdm2^C305F^ was only partial, and the selective erythroid defect caused by Mdm2^C305F^.

Taken together, our findings with the *Mdm2*^*C305F*^ knock in mice provide *in vivo* evidence that there is a delicate balance between ribosomal proteins and MDM2 binding and the stabilization of p53 as both increased RPL11 binding as well as the inability to bind MDM2 leads to p53 stabilization, a decreased number of progenitor cells and defective erythroid maturation.

## Supporting Information

S1 Fig The competitive repopulation ability of *Mdm2*^*C305F*^ mutant bone marrow. The percentage of total cell counts derived from donor mice is plotted for granulocytes and monocytes (Gr-1 and CD11b), B lymphocytes (B220), T lymphocytes (TCR β), CD4^+^ lymphocytes (CD4), CD8^+^ lymphocytes (CD8), and from peripheral blood of irradiated recipients 4, 8, or 12 weeks after BM transplantation with 5 x 10^6^ *Mdm2*^*C305F*^ mutant Bone marrow cells (CD45.2) mixed with CD45.1 allotype-marked wild type (WT) cells in 1:1 ratio. Donor ages are 6 weeks. n = 3–4 animals per genotype.
(TIF)

S2 Fig Effects of *Mdm2*^*C305F*^ mutant on spleen histology. Prussian blue stained splenic sections (Magnification: 400x).
(TIF)
