# Supplementary figures and images for: Mice with a Mutation in the Mdm2 Gene That Interferes with MDM2/Ribosomal Protein Binding Develop a Defect in Erythropoiesis
Source: PLoS One. 2016 Apr 4;11(4):e0152263. doi: 10.1371/journal.pone.0152263 (PMC4820113; doi:10.1371/journal.pone.0152263)

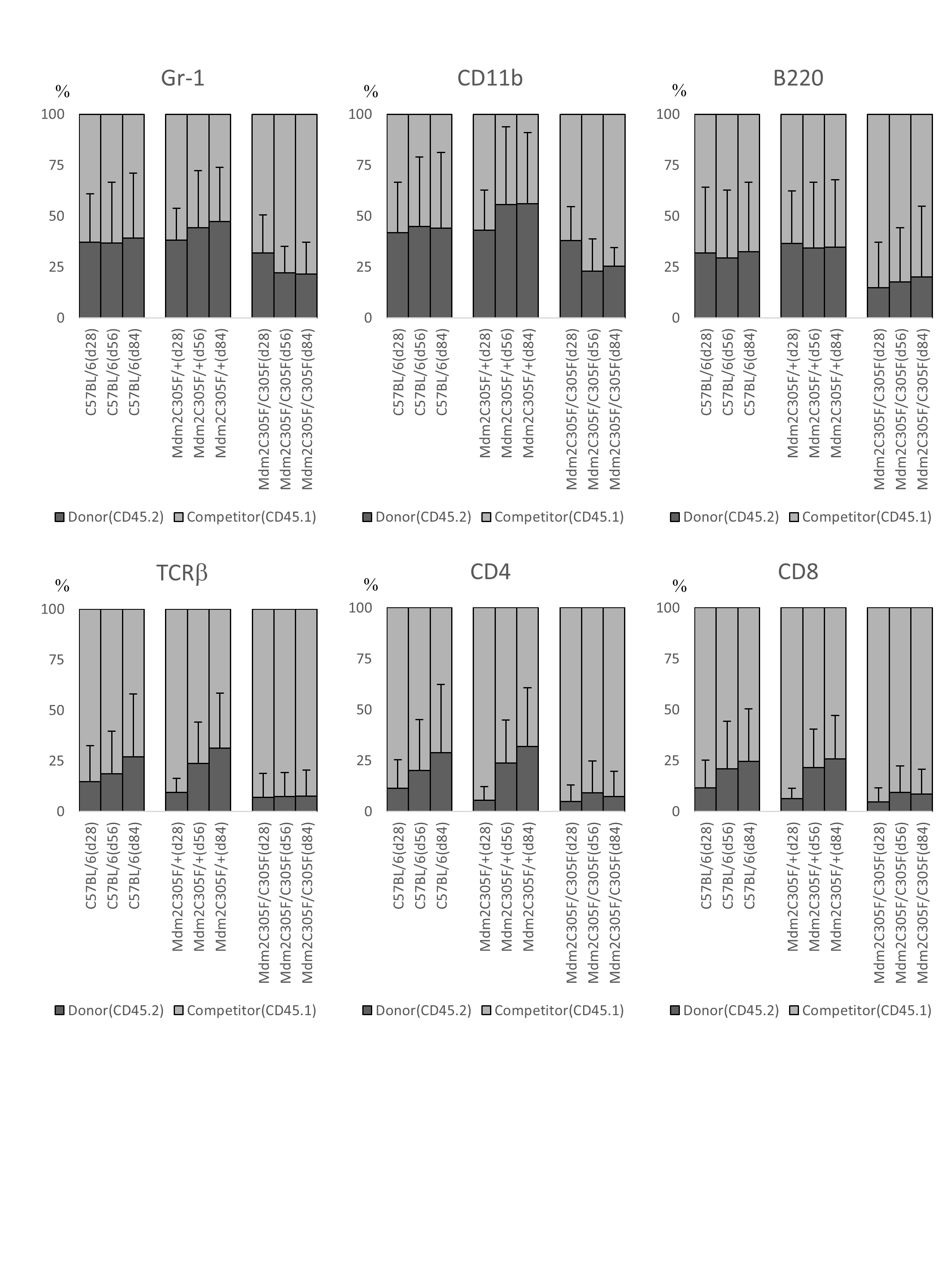

Supplement: S1 Fig — The percentage of total cell counts derived from donor mice is plotted for granulocytes and monocytes (Gr-1 and CD11b), B lymphocytes (B220), T lymphocytes (TCR β), CD4+ lymphocytes (CD4), CD8+ lymphocytes (CD8), and from peripheral blood of irradiated recipients 4, 8, or 12 weeks after BM transplantation with 5 x 10^6^ Mdm2C305F mutant Bone marrow cells (CD45.2) mixed with CD45.1 allotype-marked wild type (WT) cells in 1:1 ratio. Donor ages are 6 weeks. n = 3–4 animals per genotype. (TIF) [file pone.0152263.s001.tif]

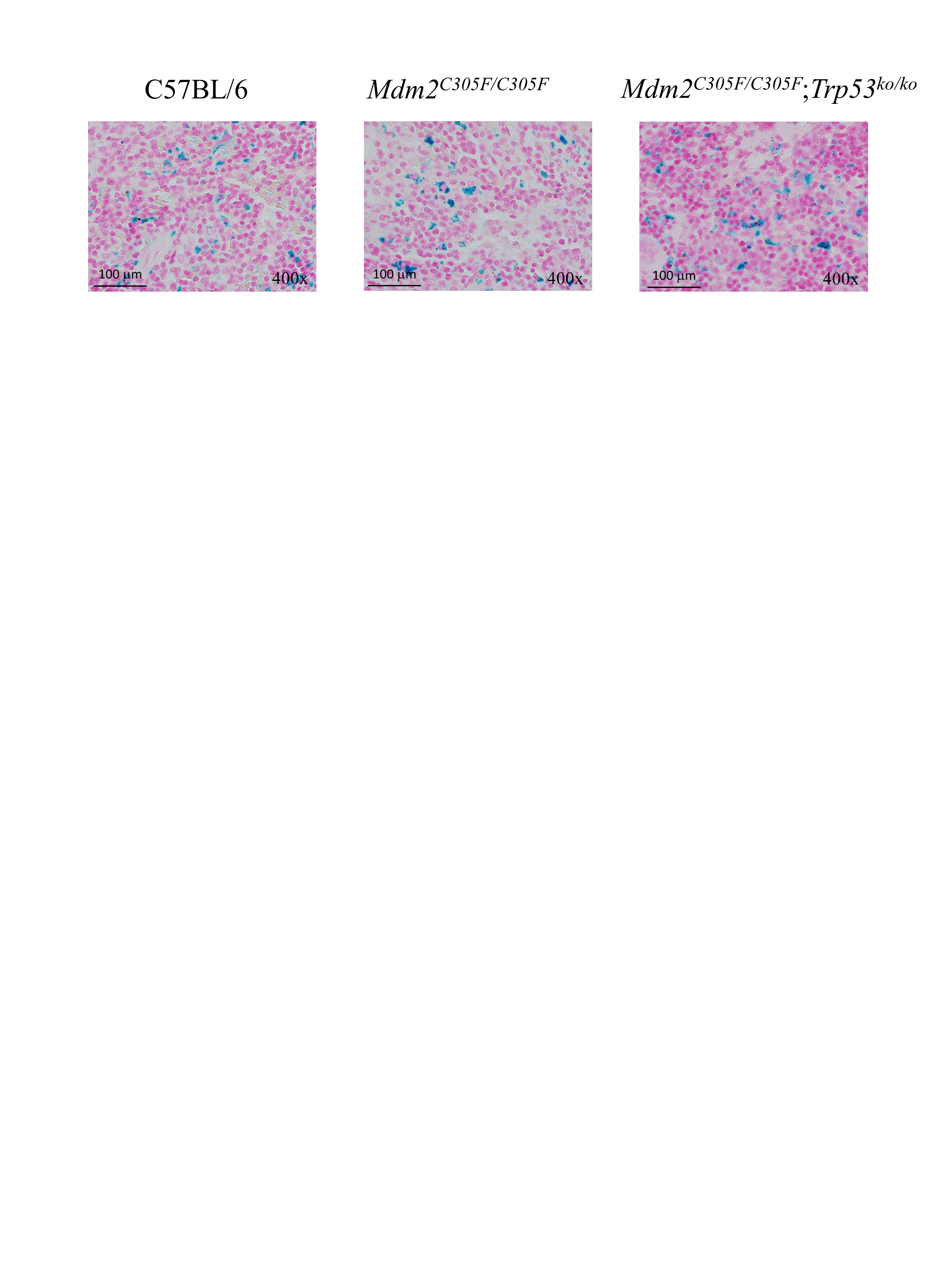

Supplement: S2 Fig — Prussian blue stained splenic sections (Magnification: 400x). (TIF) [file pone.0152263.s002.tif]
